# Q Fever in French Guiana: Tip of the Iceberg or Epidemiological Exception?

**DOI:** 10.1371/journal.pntd.0004598

**Published:** 2016-05-05

**Authors:** Loïc Epelboin, Mathieu Nacher, Aba Mahamat, Vincent Pommier de Santi, Alain Berlioz-Arthaud, Carole Eldin, Philippe Abboud, Sébastien Briolant, Emilie Mosnier, Margarete do Socorro Mendonça Gomes, Stephen G. Vreden, Magalie Pierre-Demar, Marcus Lacerda, Didier Raoult, Elba Regina Sampaio de Lemos, Félix Djossou

**Affiliations:** 1 Infectious and Tropical Diseases Department, Centre Hospitalier Andrée-Rosemon, Cayenne, French Guiana; 2 Ecosystèmes amazoniens et pathologie tropicale (EPAT), EA3593, Université de la Guyane, French Guiana; 3 Centre d’Investigation Clinique, CIC Inserm 1424, Centre Hospitalier Andrée-Rosemon, Cayenne, French Guiana; 4 Army Center of Epidemiology and Public Health, Marseille, France; 5 Direction Interarmées du Service de Santé en Guyane, Cayenne, French Guiana; 6 Institut Pasteur de la Guyane, Cayenne, French Guiana; 7 Aix-Marseille Université, Unité de Recherche sur les Maladies Infectieuses et Tropicales Emergentes, UM63, CNRS 7278, IRD 198, INSERM 1095, Marseille, France; 8 Laboratório Central de Saúde Pública do Amapá, Macapa, Amapá, Brazil; 9 Academisch Ziekenhuis Paramaribo Hospital, Paramaribo, Suriname; 10 Laboratoire Hospitalo-Universitaire de Parasitologie-Mycologie, Centre Hospitalier Andrée-Rosemon, Cayenne, French Guiana; 11 Fundação de Medicina Tropical Dr. Heitor Viera Dourado, Manaus, Amazonas, Brazil; 12 Laboratório de Hantaviroses e Rickettsioses Instituto Oswaldo Cruz/IOC—Fundação Oswaldo Cruz—FIOCRUZ, Rio de Janeiro, Brazil; Medical College of Wisconsin, UNITED STATES

## The Distribution of Knowledge and Neglect

Q fever is a cosmopolitan zoonosis caused by an intracellular bacterium, *Coxiella burnetii*. Since its discovery in 1935 in Australia, its presence has been reported almost worldwide in animals and humans [1]. In most developed countries, this infection has been widely described, and its life cycle, exposure factors, and clinical and biological pictures are well known. The incidence of Q fever is generally quite low, and most of the cases are diagnosed during short outbreaks related to direct or indirect contact of humans with cattle, sheep, or goats, which are the main reservoirs. In developing countries, information on endemicity is generally scarce and limited to seroprevalence studies in exposed populations or case reports. This presumably reflects misdiagnosis, rather than lower incidence. The diagnosis of acute Q fever mostly relies on the elevation of anti-*C*. *burnetii* antibodies by 15 to 21 days after the onset of the symptoms, detected by Immunofluorescence Assay, which is the gold standard for *C*. *burnetii* detection. However, these diagnostic techniques are often not available in tropical areas and, apparently, in numerous Latin American settings.

Indeed, an exhaustive review of the literature in English, French, Spanish, and Portuguese showed that publications on Q fever in Latin America are scarce despite the worldwide presence of the disease (Table 1). Seven countries have never reported any cases of Q fever according to the available literature (Belize, Costa Rica, Guatemala, Guyana, Honduras, Paraguay, Suriname); three haven’t reported any since 1990, but some older studies do exist (Bolivia, Panama, Venezuela); seven countries reported one or two publications since 1990 (Argentina, Chile, Ecuador, El Salvador, Peru, Trinidad, Uruguay); and Colombia, Mexico, and Brazil published several publications, including mostly case reports of chronic Q fever, one case of acute Q fever, several seroprevalence studies in exposed populations, and some studies based on an acute febrile or acute respiratory syndrome approach. Recently, Q fever was confirmed in patients and animals in parts of the Brazilian Atlantic Forest (Table 1). Thus, there are no publications on Q fever in the Amazon region except in French Guiana and Ecuador.

**Table 1:** Review of the English, Portuguese, Spanish, and French scientific literature (using the terms “Q fever” and “*Coxiella burnetii*” in MEDLINE and Google) among Q fever in South and Central America (except the Caribbean) since 1990, except French Guiana.

Country	Year of publication^1^^,^^2^	Type of study	Number of cases	Context
Argentina	2000 [2]	Retrospective descriptive study	1	One case among 408 hospitalized pneumonias in Buenos Aires
Brazil	2006 [3]	Cases series	16	Investigation among 726 febrile illness in Minas Gerais 2001–2004
Brazil	2006 [4]	Retrospective descriptive study	1	Investigation among 61 blood culture–negative endocarditis, Cardiology Hospital, São Paulo
Brazil	2008 [5]	Seroprevalence study	4/125 (3.2%)	Seroprevalence among HIV patients in Rio de Janeiro
Brazil	2008, 2011, 2012 [6–8]	Case report	3	One endocarditis in São Paulo, one chronic fever PCR positive, and one pneumonia in Rio de Janeiro
Brazil	2013 [9]	One case into a large prospective study on infective endocarditis	1	One PCR positive on surgical endocarditis, Rio de Janeiro
Brazil	2015 [10]	Longitudinal observational study	4	Study among dengue-suspected cases in Rio de Janeiro state, four Q fever cases confirmed by PCR and sequencing
Chile	2003 [11]	Seroprevalence study	36/116 (31%)	Agricultural and Livestock personal
Colombia	2006 [12]	Seroprevalence study	19/81 (23.6%)	Livestock farming individuals living in towns within Cordoba and Sucre departments
Colombia	2012, 2014 [13,14]	Case report	2	One endocarditis and one asymptomatic case in a rural man
Ecuador	2009 [15]	Longitudinal observational study	15/304	Study among acute febrile illness in the Ecuadorean Amazon Basin
El Salvador	1996 [16]	Seroprevalence study	18/40 (45%)	International study on three continents in Humans and animals
Mexico	2012 [17]	Cross-sectional pilot study	17	State of Hidalgo, rural area of central Mexico. Eight cases with clinical criteria
Mexico	1997, 2012, and 2013 [18–20]	Case reports	3	Granulomatous hepatitis
Peru	2004 [21]	Retrospective descriptive study	12/152 (9%)	Outbreak of febrile illness in 2002 in the district of Sapillica
Trinidad	2011 [22]	Seroprevalence study	20/455 (4.4%)	Livestock and abattoir workers
Uruguay	1994 [23]	Case report	1	Endocarditis

^1^ Existing publications before 1990, but none since then: Bolivia, Panama, Uruguay, Venezuela

^2^ No publication found at all: Belize, Chile, Guyana, Honduras, Suriname

## Q Fever in Travellers and Migrants Returning from Latin America

Q fever is a rare disease in travellers, especially those returning from Latin America. Although Suriname reported no cases, one case of myocarditis due to *C*. *burnetii* was diagnosed in the Netherlands in an 8-year-old child whose father had recently returned from Suriname [24]. Furthermore, a seroprevalence study in the same country showed that *C*. *burnetii* antibodies positivity was associated with being from Suriname, Turkey, or Morocco [25]. A case of Q fever was reported in Spain in a traveller returning from 15 days of travel in the Dominican Republic and Venezuela [26]. Several cases of *C*. *burnetii* pneumonia were reported in travellers returning from French Guiana [27]. Recently, the French National Centre for Rickettsiosis in Marseille described genotypes of Q fever according to the presumed infection area. No case was reported in patients returning from South America, except for French Guiana.

## The Singular Epidemiology of Q Fever in French Guiana

French Guiana is a French overseas territory located on the northeastern coast of South America. About 90% of its 84,000 km^2^ surface is covered by the Amazonian rainforest; the remaining 10%, located in the north, consists of a coastal plain where 90% of the 250,000 inhabitants live. Almost half of the population lives in Cayenne. It is an outermost region of the European Union, with technical and financial resources that are closer to European countries than to the neighbouring countries in the fields of health and research.

*C*. *burnetii* was first described in 1955 in French Guiana, but the real interest arose in 1998 when three severe cases were described [28]. Antibodies to *C*. *burnetii* were tested among 275 stored samples from patients tested for dengue fever from 1992 to 1996: 9.1% were positive with a sharp increase in 1996 (23.9%). The seroprevalence was much higher in Cayenne than in rural areas. Subsequent studies found an annual incidence of 37 cases/100,000 persons between 1996–2000, up to 150 cases/100,000 persons in 2005 [29], and 17.5/100,000 persons between 2008 and 2011 [30]. *C*. *burnetii* primary infection is also more frequently symptomatic, with more patients presenting with fever in Cayenne compared to Metropolitan France (97% versus 81% in Marseille, *p* < 0.0001) [30]. While pneumonias only represent 8% to 37% of symptomatic Q fever in France [30], they account for about 90% of the cases in French Guiana [29,30]. While *C*. *burnetii* is the causal pathogen for about 1% of cases of community-acquired pneumonia requiring hospitalization in the United Kingdom and continental Europe, 2.3% in North America, and 5.8% in Israel, a highly endemic region [31], it is implicated in 24% to 38% of pneumonias in the area of Cayenne [32], which is the highest prevalence ever described worldwide. Consequently, the empirical antibiotherapy for community-acquired pneumonia in Cayenne is comprised of doxycycline in order to treat *C*.*burnetii*. Also, the initial presentation of *C*.*burnetii* pneumonia in Cayenne is severe, with more frequent symptoms like chills, headache, night sweats, and arthromyalgia than pneumonias from other aetiologies [32]. This high rate of symptomatic *C*. *burnetii* primary infection has a significant public health impact. Regarding persistent focalized infections, the incidence of *C*. *burnetii* endocarditis is the same in Cayenne as in Metropolitan France [30], and further studies are needed to assess the prevalence of endocarditis and vascular infections by *C*. *burnetii*, which are very severe diseases that are probably underestimated in this territory. The strategy of screening for risk factors for endocarditis (valulopathy and valvular prosthesis) by systematic echocardiography is the same as the one recommended in Metropolitan France. If a risk factor is detected, a prophylactic treatment (doxycycline and hydroxychloroquine) should be initiated because it has proven its efficacy in reducing the incidence of such infections [33].

*C*. *burnetii* epidemiology in French Guiana remains unclear: groups at risk are not clearly defined, and the classical risk factors are not observed, especially professional exposure to cattle. The main risk factors for *C*. *burnetii* infection are working in construction/public works, living near bats, wild mammals, or the forest, levelling work, and gardening [29]. Surprisingly, French expatriates were more frequently infected than people from other communities in French Guiana. The hypothesized reservoir remains currently controversial. Several studies have tested bats, cattle, sheep, goats, small mammals, domestic mammals, and birds, in vain [29,34]. Recently, the three-toed sloth (*Bradypus tridactylus*) has been incriminated as a possible reservoir of the bacterium in Cayenne. *C*. *burnetii* MST 17 has been detected in the spleen, stools, and ticks of a dead sloth near a recent outbreak site [34]. In addition, Q fever incidence was correlated with three-toed sloth birth numbers 1–2 months before, peaking during the rainy season in French Guiana [35]. However, for many animal species in French Guiana reproduction is related to the rainy season.

Although the role of the three-toed sloth in transmission is an interesting hypothesis to explore, it is probably not the only reservoir and seems unlikely to be the sole explanation for the magnitude of this problem in French Guiana. Another particularity of Q fever in French Guiana is that all the cases identified with Polymerase Chain Reaction (PCR) were due to the genotype MST 17 [36], isolated specifically from eight patients having travelled to or lived in Cayenne. Conversely, it was not detected in any of the 298 strains of *C*. *burnetii* from other geographical areas [36]. This unique MST 17 clone provokes an exceptional, strong immune response with very high levels of phase I IgG in the acute phase of the disease [30]. It is also more virulent, as illustrated by the high prevalence of Q fever pneumonia in French Guiana and the more severe initial presentation than pneumonias of other aetiologies [32,37]. Recently, an MST 17 strain (*C*. *burnetii* 175) was sequenced and revealed a unique feature: a 6105 bp-deletion in the *hlyCABD* operon of the Type 1 Secretion System (T1SS). This deletion has been detected by qPCR in eight other MST 17 strains and in none of the 298 strains of the French National Referral Centre database [38]. The genome reduction observed in the MST 17 clone is possibly linked to its exceptional pathogenicity and emergence in Cayenne.

## Local Emergence or Widespread Neglect?

Q fever is supposed to be well known and cosmopolitan. Nevertheless, the contrast between the high incidence and prevalence among pneumonias in French Guiana and the near absence of data in neighbouring countries is intriguing. It may be simply due to circumscribed emergence. However, this raises the question of the underdiagnosis of *C*. *burnetii* infections due to lack of diagnostic tests and the lack of awareness by physicians in the Amazonian region, where no cases were reported. This infection should be found in surrounding countries, as infectious agents are not contained by borders. Several cases of acute Q fever are diagnosed in Europe in travellers returning from the countries of the Amazon, and only endocarditis and severe cases are published in the Brazilian medical literature (Table 1). Thus, these cases may be considered as the tip of the iceberg. Although at this point estimates are speculative, the potential incidence of Q fever in French Guiana could be 17.5 to 150/100,000 inhabitants per year. Based on this estimate and assuming similar incidence in countries with similar fauna in the Guiana Shield (Guyana, Suriname, French Guiana, and Amapá combined have approximately 2,230,000 inhabitants), there may be 440 to 3,330 undiagnosed cases per year. Expanding this to the Amazonian region, including northern regions of Brazil (Acre, Rondônia, Para, Roraima, Amazonas, and Tocantins combined have approximately 17,423,343 inhabitants), estimated cases might be 2,960 to 26,135 cases a year. These computations of the potential burden of Q fever are estimates with incomplete data and don’t include populations of the Amazonian areas of Colombia, Venezuela, Ecuador, Bolivia, and Peru.

It is difficult to believe that *C*. *burnetii* would limit its spread beyond the borders of French Guiana. This apparent “emergence” in the territory with the highest GDP per capita of the South American continent, thus with the highest diagnostic resources, suggests that a plausible explanation of the gap of cases of Q fever in most of the Amazonian part of South America is one of a vicious cycle in which a lack of diagnostic tools leads to lack of evidence from diagnostic algorithms, perpetuating the lack of diagnostic tools. It is nevertheless possible that other countries in the Amazon region do not have a high incidence of Q fever. Indeed, Nova Scotia in the 1980s had very high rates of Q fever [39], but these rates were never seen elsewhere in Canada. Ultimately, studies need to be done to test this point.

The many singularities of Q fever in French Guiana warrant further studies throughout the Amazon, such as prospective studies among fevers of unknown origin, with a special focus on community-acquired pneumonia, and molecular studies on wild animal reservoirs and transmission. Better diagnostic techniques and rapid diagnostic tests, routine PCR, better surveillance systems, and intensified international collaboration are needed to map the true burden of Q fever in Latin America. This knowledge would then help to adapt treatment protocols of pneumonia and avoid the chronic consequences of Q fever that may develop when adequate treatment is not given. These investigations will help to propose adapted screening, prophylaxis, and treatment strategies for Q fever in this region.
